# Antidiabetic Potential of Volatile Cinnamon Oil: A Review and Exploration of Mechanisms Using In Silico Molecular Docking Simulations

**DOI:** 10.3390/molecules27030853

**Published:** 2022-01-27

**Authors:** Nicole Stevens, Kathryn Allred

**Affiliations:** dōTERRA International, Pleasant Grove, UT 84062, USA; nstevens@doterra.com

**Keywords:** cinnamon, cinnamon essential oil, molecular docking, antidiabetic, glucose metabolism, cinnamaldehyde, beta-caryophyllene, eugenol

## Abstract

Cinnamon has been used as a flavoring and medicinal agent for centuries. Much research has focused on cinnamon bark powder, which contains antioxidants, flavonoids, carotenoids, vitamins, minerals, fiber, and small amounts of essential oil. However, isolated and concentrated cinnamon essential oil may also have important medicinal qualities, particularly in antidiabetic therapy. Some of the most common essential oil constituents identified in the literature include cinnamaldehyde, eugenol, and beta-caryophyllene. Due to their high concentration in cinnamon essential oil, these constituents are hypothesized to have the most significant physiological activity. Here, we present a brief review of literature on cinnamon oil and its constituents as they relate to glucose metabolism and diabetic pathogenesis. We also present molecular docking simulations of these cinnamon essential oil constituents (cinnamaldehyde, eugenol, beta-caryophyllene) that suggest interaction with several key enzymes in glucometabolic pathways.

## 1. Introduction

Essential oils are complex mixtures of small, aromatic molecules produced by many plant taxa as secondary metabolites related to normal metabolic processes [1,2]. The diverse composition of these secondary metabolites is thought to reflect both genetic species’ individuality as well as an adaptation mechanism for plants to cope with environmental stressors such as pathogens, unfavorable temperature and pH, drought, high salinity, ultraviolet radiation, heavy-metal stress, and nutrient depletion [2,3]. Additionally, these molecules may be used by the plant for defense against herbivores and as an attractant for pollinators. Essential oil compounds are stored in microscopic sacs or glands in various parts of the plant.

Essential oil terpenoids are based on a basic isoprene unit: five carbons, (two of which are double-bonded) and eight hydrogens. Monoterpenes consist of two isoprene units bonded together (10 carbons), and sesquiterpenes are three isoprene units (15 carbons). Occasionally, heavier diterpene (4 isoprene units, 20 carbons) molecules can be represented in cold-pressed or solvent-extracted essential oils, but these molecules tend to be too heavy to be carried over by the steam during typical distillation. Monoterpenes and sesquiterpenes can either be straight-chain (acyclic) or contain various ring structures (monocyclic, bicyclic, etc.), and there is considerable variety in these structures. Most volatile essential oil compounds are produced along canonical biochemical pathways (see Table 1) from a variety of precursor molecules, including amino acids, carbohydrates, and fatty acids and their derivatives [4,5].

Some essential oils have been studied for their antidiabetic potential [6,7,8,9,10]. The US Centers for Disease Control reports that about 34.2 million people (around 10% of the total population) in the United States have diabetes, and about one in five of those people do not realize they have the disease [11]. Diabetics are at much higher risk of early mortality as well as comorbidities such as blindness, renal disease, heart disease, stroke, and lower-limb amputations [12]. New drugs based on complex mixtures of natural compounds may offer therapeutic potential for treatment of diabetes.

Understanding the interaction between small molecules and possible enzyme binding sites is key in discovering novel therapeutics [13]. The field of In silico modeling is becoming an important player in early-stage drug discovery to suggest possible interactions between ligands and proteins of interest, as well as predict the relative strength of those interactions.

The goal of this article is two-fold: (1) outline existing literature on anti-diabetic potential of cinnamon essential oil and its main constituents; and (2) through molecular docking simulations, suggest possible mechanisms of glucose metabolism modulation by cinnamon essential oil.

## 2. Cinnamon Essential Oil and Its Constituents

Cinnamon has been used as a flavoring and medicinal agent for centuries, with references back to Biblical times [14]. Medicinally, cinnamon has been used as a carminative and treatment for digestive issues such as dyspepsia, anorexia, and vomiting [15]. Other anecdotal uses of cinnamon include treatment for hemorrhage and bleeding ulcers [16], as a warming agent and stimulant [17], and treatment against various cancers [18]. Modern investigations have focused primarily on cinnamon’s antidiabetic and antilipidemic properties, although the therapeutic benefit and dosing parameters require further exploration [19]. Much of this research involves cinnamon bark powder, which contains small amounts of cinnamon essential oil along with antioxidants, flavonoids, carotenoids, vitamins, minerals, and fiber. However, concentrated, volatile cinnamon essential oil may also have important medicinal qualities.

There may be more than 200 *Cinnamomum* species, but several species of cinnamon have been identified as culturally or economically relevant: *Cinnamomum zeylanicum* (botanical synonym: *C. verum*, also called Ceylon cinnamon or true cinnamon), *C. cassia*, *C. burmannii*, *C. tamala*, *C. loureirii*, *C. iners*, *C. pauciflorum*, *C. camphora* and *C. glaucescens* [20]. Each species yields an essential oil of different chemistry and aroma. Only *C. zeylanicum* (Ceylon cinnamon) and *C. cassia* are currently produced on a commercial scale.

*Cinnamomum zeylanicum* trees are slender evergreens that can reach up to 65 feet in height. Cinnamon bark is harvested from the inner cambium of trees typically 3–4 years old. Trees are pruned repeatedly in order to curtail height for easier harvesting and encourage lateral growth of shoots suitable for collection [14,21]. Curls of bark (called quills) are cut from the tree and allowed to dry. Essential oil sacs are located within the cambium and tend to be in the range of 2–10 microns in diameter. Essential oil yield is about 1–4% from the cinnamon bark.

Cinnamon essential oil is a volatile, hydrophobic, mildly viscous liquid, usually yellowish in color. It has a sweet, spicy, woody aroma. The volatile oil is typically isolated by hydrodistillation from bark or leaves of cinnamon trees [22]. Physical characteristics are represented in Table 2. Both monoterpenes and sesquiterpenes, and their functional variations, are present in cinnamon essential oil. Several important cinnamon essential oil constituents identified in the literature include cinnamaldehyde, eugenol, and beta-caryophyllene [23]. These constituents are usually present in appreciable amounts in cinnamon oil, and will be the focus of this paper.

Cinnamaldehyde (3-phenylprop-2-enal) is a phenylpropanoid naturally synthesized along the shikimate pathway in plants from the precursor amino acid phenylalanine [26]. The molecule has the chemical formula C_9_H_8_O and contains a benzene ring, a short carbon chain, and an aldehyde group [27]. Both cis- and trans-cinnamaldehyde conformations exist in nature, but the majority of cinnamaldehyde in cinnamon bark tends to be the trans- stereoisomer. This constituent of cinnamon oil represents approximately 50–90% of total chemical makeup, depending on the species and plant part from which it was derived. Cinnamaldehyde is the primary contributor of the sweet, spicy aroma typically associated with cinnamon essential oil. However, as is often the case, multiple constituents contribute to the rich and complex aroma that distinguishes whole cinnamon bark essential oil from isolated cinnamaldehyde [28].

Eugenol (4-allyl-2-methoxyphenol) is a phenylpropanoid derived from guaiacol with an allyl chain substitution [29]. It is a member of the phenol class of compounds, synthesized along the shikimate pathway from the precursor amino acid phenylalanine. The molecule has the chemical formula C_10_H_12_O_2_ and contains a benzene ring, with a hydroxy group and an ether group in ortho-conformation and the allyl chain para to the hydroxy group. Eugenol is the main constituent of essential oil derived from cinnamon leaf, while in essential oil derived from bark, it is typically present in about 2–13% (see Table 3).

Beta-caryophyllene, or (-)-trans-caryophyllene, is a bicyclic sesquiterpene found in several plants used for spice and flavoring, including cinnamon [30]. This molecule is produced along the mevalonate pathway in plants from precursor molecules of acetyl-CoA. In nature, beta-caryophyllene occurs along with alpha-humulene (formerly called alpha-caryophyllene), which has a ring-opened structure. Due both to its unique nine-membered double-ring structure, which is uncommon in nature, as well as its desirable sweet aroma and taste, beta-caryophyllene has attracted attention as a subject of research. In cinnamon oil, beta-caryophyllene is typically present at less than 10%.

## 3. Literature Review of Antidiabetic Properties of Cinnamon Essential Oil and Its Constituents

Table 4 outlines representative publications that specifically study the effects of cinnamon essential oil and its main chemical constituents on glucose metabolism pathways and diabetic endpoints. This review is not comprehensive for all preparations or uses of cinnamon. Specifically, we review studies that include distilled or hydrodistilled cinnamon volatile oil or isolated cinnamon oil constituents (specifically, cinnamaldehyde, eugenol, and beta-caryophyllene) for antidiabetic potential.

Studies on cinnamon essential oil demonstrate improvement of fasting blood glucose, fasting insulin, and improvement in both anatomy and function of kidney and liver cells. Improvement in enzyme function, both enzymes involved directly in glucose metabolism and enzymes involved in excretion, was also consistently noted. Studies including cinnamaldehyde also showed improvement of fasting blood glucose, increased insulin sensitivity, decreased appetite, and both up- and down-regulation of myriad proteins associated with glucose metabolism.

While most current in vitro and in vivo research focuses on cinnamaldehyde as the primary therapeutic agent in cinnamon oil, other constituents may also play a supporting role in this oil’s observed antidiabetic properties. Eugenol and beta-caryophyllene both show promising therapeutic benefits when tested in animal models. Research outlined in Table 4 suggests these constituents may function by enhancing native antioxidant systems and ameliorating oxidative damaged caused by development of diabetes.

Research to date suggests that cinnamon essential oil may have therapeutic benefit in modulating glucose metabolism along multiple pathways. Additional in vitro and in vivo studies are needed (particularly human clinical trials) to fully elucidate the activity and mechanisms of cinnamon oil’s potential antidiabetic effect.

Next, using In silico molecular modeling, we attempt to predict possible enzymatic mechanisms through which main constituents of cinnamon essential oil may exert effects. Models will also provide estimated binding affinities that forecast the likelihood of spontaneous interaction between ligand and target. These models offer an initial framework for further in vitro and in vivo validation.

## 4. In Silico Docking Models

**Figure 1:**
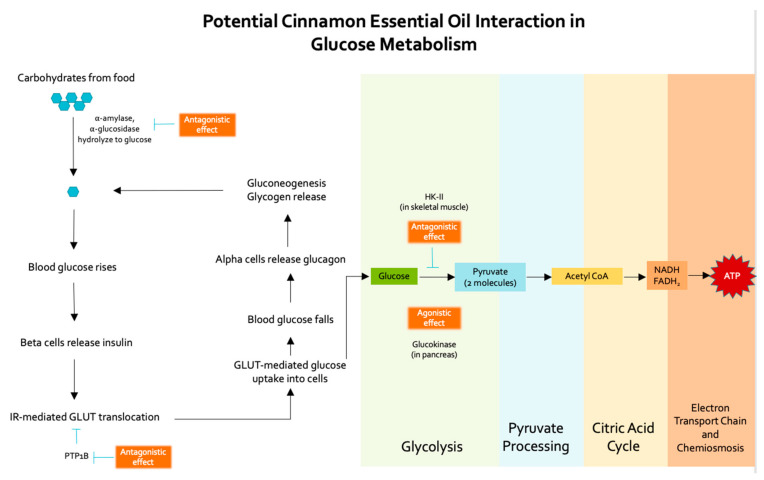
Hypothesized interactions of cinnamon essential oil constituents with enzymes along glucose metabolism pathways.

Three cinnamon essential oil constituents, cinnamaldehyde, eugenol, and beta-caryophyllene, combined comprise 60–70% of cinnamon essential oil. As suggested by the literature and because of their concentration in oil, these constituents are hypothesized to have the most significant physiological effects. Thus, cinnamaldehyde, eugenol, and beta-caryophyllene were selected for molecular docking analysis and simulated against enzymes involved in glucose metabolism or the glycolysis pathway (glucokinase, alpha-amylase, PTP1B, alpha-glucosidase, and hexokinase-II (HK-II). Hypothesized interactions of essential oil constituents with these glucose metabolism enzymes are represented in Figure 1.

As interactions with small molecules can result in a variety of enzymatic responses, it is important to note the overall effect of the binding model. Most commonly, a xenobiotic substance can be:

- Agonistic: behaving like the natural ligand, agonist molecules bind to the receptor and trigger signaling. When a small molecule is simulated to bind in the same active site as a drug or mimetic with known biological effects, it is considered an agonist.
- Antagonistic: inhibiting the effect of the natural ligand or agonist by blocking the active site of the enzyme (competitive agonist), by binding elsewhere on the enzyme and altering the biological function (noncompetitive antagonist), or by covalently altering the binding site (irreversible agonist). A small molecule binding in this manner would inhibit the activity of the enzyme and alter downstream pathway outcomes.

In the case of glucose metabolism enzymes, both agonistic and antagonistic activities may be useful therapeutics for treating Type II Diabetes. In alpha-amylase and alpha-glucosidase, antagonistic effects from essential oil constituents are desirable; specifically, small molecules bind in the active site and block the binding and subsequent hydrolysis of the physiologic ligands (complex carbohydrates). Essential oil constituents shown to interact at this site may serve to slow the rise in blood glucose following food intake.

In PTP1B, antagonistic effects from essential oil constituents are also desirable. Specifically, small molecules bind in the active site and block the binding of the natural ligand (phosphate group on insulin receptors [70]). This effectively inhibits the PTP1B enzyme, allowing the insulin receptor to remain phosphorylated, and ultimately allowing increased GLUT4 translocation to the cell surface. If essential oil constituents bind at this site, lower blood glucose levels may result as a higher volume of glucose molecules is cleared from the blood through uptake by GLUT4.

In glucokinase, the natural ligand (glucose) is converted to glucose-6-phosphate as the initial step in glycolysis, and this process is an important regulator in insulin release and glucose metabolism [71]. Glucokinase serves as both a glucose sensor in pancreatic beta-cells and as a rate-controlling enzyme for glycogen synthesis and hepatic glucose clearance [72]. For this enzyme, activation is hypothesized to occur through ligand binding at an allosteric site rather than the active site. This allows the active site to remain available for glucose metabolism. Small molecules, such as essential oil constituents, that can bind in the allosteric pocket could serve glucokinase agonists, increasing glucose metabolism and allowing for more sensitive glucose homeostasis.

Hexokinases catalyze the conversion of glucose to glucose-6-phosphate, the first step in many glucose metabolism pathways. Overexpression of hexokinase is common in certain cancers due to its ability to reduce serum levels of glucose, insulin, and insulin-like growth factor [73]. Antagonistic reduction in hexokinase activity has therapeutic implications for diabetes as well; the drug metformin works in part by reducing activity of hexokinase. HK-II is the predominant form found in skeletal muscle and is insulin-dependent. Recently, a compound called benserazide has demonstrated ability to selectively inhibit HK-II [74]; other compounds binding in the same site as HK-II antagonists could provide new therapeutic targets for diabetes.

## 5. Materials and Methods

### Molecular Docking

In silico molecular docking simulations were performed using the AutoDock Vina [75] (Scripps Research, La Jolla, CA, USA, version 1.1.2) module within UCSF Chimera [76] (University of California, San Francisco, CA, USA, version 1.13.1). Protein crystal structures were downloaded from Protein Data Bank (www.rcsb.org (accessed on 3 December 2021)). Criteria for crystal structure selection included unmutated proteins from *Homo sapiens* with complete structure representation and resolution less than or equal to 3 angstroms. Protein models were prepared for docking using the Mac Command Line interface and the Dock Prep tool in UCSF Chimera. Docked models were qualitatively visualized in UCSF Chimera (1.13.1). Ligand structures were downloaded from MolView (molview.org (accessed on 2 December 2021)) prepared for docking within UCSF Chimera.

Modeled enzymes included: alpha-amylase, alpha-glucosidase, glucokinase, PTP1B, and HK-II (PDB ID: 1HNY, 3TOP, 5V4W, 1BZJ, and 2NZT, respectively [77,78,79,80,81]). Simulations were run with essential oil constituents cinnamaldehyde, eugenol, and beta-caryophyllene. Positive controls included known antagonist acarbose for alpha-amylase and alpha-glucosidase, known glucokinase agonist piragliatin, known PTP1B antagonist TPICOOH, and known HK-II antagonist, benserazide [74].

## 6. Modeling Results and Discussion

Models were initially evaluated by strength of predicted interactions. Proteins were probed for interaction with essential oil constituents or a positive control both at active sites and at possible allosteric binding locations. Binding affinities that were estimated to have a change in Gibbs free energy (ΔG) of −6.0 kcal/mol or more negative were considered to represent activity through spontaneous physiologic interaction [82].

All simulated binding affinities are represented in Table 5. Overall, beta-caryophyllene showed the highest binding affinity across multiple proteins. Cinnamaldehyde and eugenol also showed potential interaction with select proteins.

**Figure 2:**
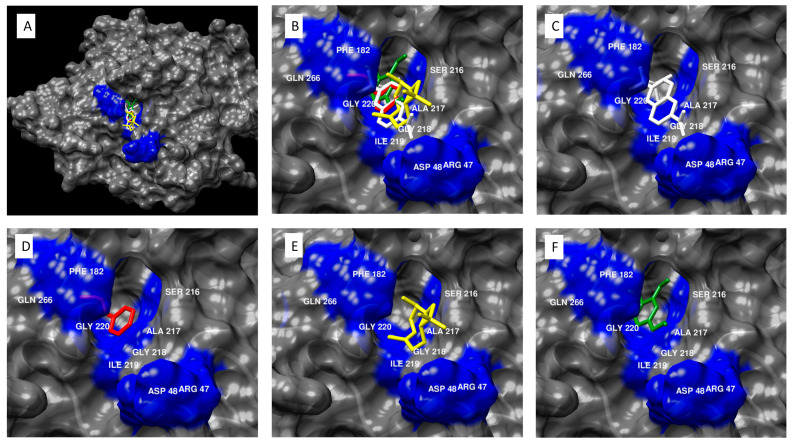
Molecular modeling of cinnamon essential oil constituents docked with PTP1B. (**A**) PTP1B antagonist TPICOOH and essential oil compounds cinnamaldehyde, beta-caryophyllene, and eugenol docked in the active site of PTB1B. (**B**) Close view of PTB1B antagonist TPICOOH and essential oil compounds cinnamaldehyde, beta-caryophyllene, and eugenol docked in the active site of PTB1B, specifically, near residues ARG47, ASP48, PHE182, SER216, ALA217, GLY218, ILE219, GLY220, ARG221, and GLN266. (**C**) TPICOOH docked in the active pocket of PTB1B, with a binding affinity of −9.7 kcal/mol. (**D**) Cinnamaldehyde docked in the active pocket of PTB1B, with a binding affinity of −6.5 kcal/mol. (**E**) Beta-caryophyllene docked in the active pocket of PTB1B, with a binding affinity of −5.6 kcal/mol. (**F**) Eugenol docked in the active pocket of PTB1B, with a binding affinity of −6.0 kcal/mol.

The PTP1B active site includes the following residues (Figure 2): ARG 47, ASP 48, PHE 182, SER 216, ALA 217, GLY 218, ILE 219, GLY 220, ARG 221, and GLN 266 [80]. Binding in the active site of PTP1B serves to inhibit the enzyme (which itself inhibits the insulin receptor-mediated GLUT4 translocation to the membrane). Cinnamaldehyde and eugenol from cinnamon essential oil showed binding affinity for the PTP1B active site. These constituents demonstrated binding affinities stronger than −6.0 kcal/mol, indicating a high likelihood that the binding reaction will occur spontaneously. While none of the modeled essential oil constituents demonstrated binding affinities as strong as the positive control, known PTP1B antagonist TPICOOH, it is interesting to consider the implications of an essential oil with broad chemistry that may be able to interact along multiple points of a pathway and with several different constituents.

**Figure 3:**
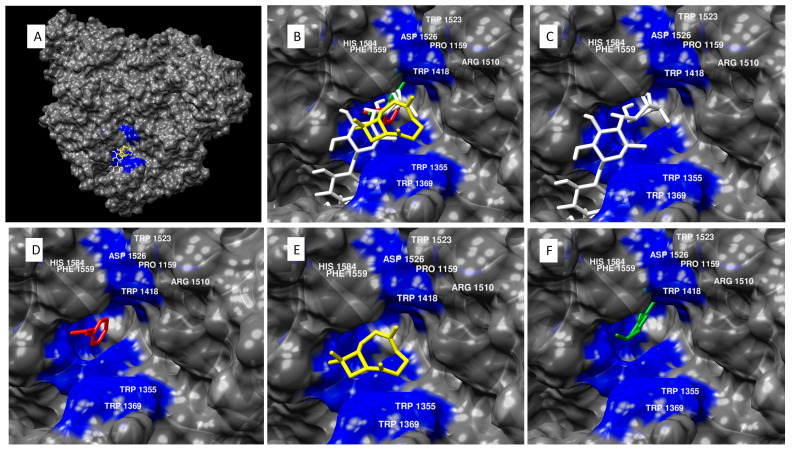
Molecular modeling of cinnamon essential oil constituents docked with alpha-glucosidase. (**A**) Alpha-glucosidase antagonist acarbose and essential oil compounds cinnamaldehyde, beta-caryophyllene, and eugenol docked in the active site of alpha-glucosidase. (**B**) Close view of alpha-glucosidase antagonist acarbose and essential oil compounds cinnamaldehyde, beta-caryophyllene, and eugenol docked in the active site of alpha-glucosidase, specifically, near residues HIS 1584, TRP 1523, TRP 1418, ARG 1510, TRP 1355, PHE 1559, ASP 1526, TRP 1369, and PRO 1159. (**C**) Acarbose docked in the active pocket of alpha-glucosidase, with a binding affinity of −8.3 kcal/mol. (**D**) Cinnamaldehyde docked in the active pocket of alpha-glucosidase, with a binding affinity of −6.1 kcal/mol. (**E**) Beta-caryophyllene docked in the active pocket of alpha-glucosidase, with a binding affinity of −6.6 kcal/mol. (**F**) Eugenol docked in the active pocket of alpha-glucosidase, with a binding affinity of −6.7 kcal/mol.

Alpha-glucosidase inhibition takes place at the active pocket (Figure 3), which includes the following residues: HIS 1584, ASP 1279, TYR 1251, TRP 1523, TRP 1418, ARG 1510, TRP 1355, PHE 1559, ASP 1526, TRP 1369, and PRO 1159 [78]. Acarbose, a known alpha-glucosidase antagonist and frontline drug in diabetic therapy [83], was shown to bind in the same pocket. Once again, two of the main constituents from cinnamon essential oil (cinnamaldehyde and eugenol) showed △G more negative than −6.0 kcal/mol.

The active site of the alpha-amylase enzyme includes the following residues: ASP 197, GLU223, and ASP300 [77] (see Supplemental Materials, Figure S1). Beta-caryophyllene demonstrated notable binding affinity in this active site, at −6.9 kcal/mol, especially considering acarbose interacted at −7.3 kcal/mol at the same site. Cinnamaldehyde and eugenol did not demonstrate significant binding affinity.

For glucokinase binding simulations, essential oil constituents were modeled against the positive control piragliatin, a relatively new glucokinase agonist studied in patients with Type II Diabetes [84,85,86]. All constituents showed qualitative binding in an allosteric site bordered by residues VAL 62, ARG 63, TRP 99, TYR 214, MET 235, LYS 458, LYS 459, and LEU 451 (see Supplemental Materials, Figure S2). This is consistent with modeling conducted by Liu et al. [87], who noted that all known glucokinase agonists bind at the same allosteric site. None of the constituents bound as strongly as the positive control (△G = −9.5 kcal/mol); however, beta-caryophyllene and eugenol demonstrated relatively strong binding affinity (△G = −6.3 and −6.0 kcal/mol, respectively).

In a previous publication, the compound benserazide showed strong inhibition of HK-II, interacting with residues GLY 681, THR 680, and ASN 656 [74] (see Supplemental Materials, Figure S3). In our simulations, benserazide interacted at this site with a binding affinity of −7.4 kcal/mol. Beta-caryophyllene demonstrated comparatively strong binding affinity of −6.6 kcal/mol, while the other essential oil constituents all scored greater than −6.0 kcal/mol. However, beta-caryophyllene and cinnamaldehyde interacted at a different site than the control, so additional in vitro testing is needed to elucidate whether this interaction translates into modulation of enzymatic activity.

## 7. Limitations

In silico modeling does not replace in vitro and in vivo work; while it predicts possible mechanisms of interaction between essential oil constituents and enzymes of interest, these must be confirmed through additional testing. Modeling software may incorrectly predict binding due to underlying assumptions or errors in protein crystal structure determination. In addition, although docking simulations, in vitro, and in vivo studies all point toward therapeutic antidiabetic activities of cinnamon essential oil and some of its main constituents, high-quality clinical studies are needed to confirm these activities in complex human systems.

## 8. Conclusions

In vitro, in vivo, and In silico testing indicate that volatile cinnamon oil and some of its main constituents possess antidiabetic properties along different pathways. As reviewed here, numerous studies validate cinnamon oil’s antioxidant properties and suggest that the reduction in oxidative stress is one mechanism through which it may exert antidiabetic effects. In silico molecular docking simulations suggest that constituents in cinnamon essential oil may affect multiple enzymes along glucose metabolism pathways, including alpha-glucosidase, alpha-amylase, PTP1B, glucokinase, and HK-II. While none of the modeled essential oil constituents demonstrated binding affinities as strong as their comparator drugs, they may be able to exert diverse effects across multiple metabolic pathways, potentially increasing net therapeutic benefit. To our knowledge, this is the first In silico study of this set of glucose metabolism enzymes and their theoretical interactions with volatile cinnamon oil constituents, highlighting their antidiabetic potential.

As additional studies are conducted, particularly human clinical trials, the potential therapeutic benefits of volatile cinnamon compounds will be further evaluated. Although cinnamon oil has been part of the human diet in small amounts for millennia and is considered relatively safe, critical safety studies must also be conducted to determine the feasibility of using volatile cinnamon compounds in chronic, therapeutic modalities.

## Figures and Tables

**Table 1:** Canonical Biochemical Pathways for Production of Volatile Compounds in Plants.

Biochemical Pathway	Classes of Compounds Produced	Examples
Mevalonate	Terpenes and terpenoids	Beta-caryophyllene, limonene, pinenes, geraniol
Non-mevalonate, also called methylerythritol phosphate (MEP)		
Shikimate	Alkaloids, phenylpropanoids, flavonoids, lignans, aromatic polyketides	Cinnamaldehyde, eugenol, coumarin
Derivation of fatty acids	Fatty alcohols	Octanol, decanol

**Table 2:** Physical properties of cinnamon essential oil [24,25].

Cinnamon Essential Oil		
Physical Property	Typical Value	Range
Optical Rotation	−1	−20–+20
Specific Gravity	1.02	1.01–1.07
Refractive Index	1.59	1.53–1.61
Flash Point	87 °C	62–104 °C
Boiling Point	248 °C	248–249 °C
Color	Dark yellow	Yellow to light brown
Aroma	Spicy, sweet	Spicy, sharp, woody

**Table 3:** Comparative constituents of several cinnamon species. Common constituents in cinnamon essential oil extracted from various plant parts of *Cinnamomum zeylanicum* and *Cinnamomum cassia* [22,23,31,32,33,34].

*C. zeylanicum* Bark	Composition	*C. zeylanicum* Leaf	Composition	*C. cassia* Bark	Composition	*C. cassia* Leaf	Composition
(E)-Cinnamaldehyde	44.2–75.7%	Eugenol	68.6–87.0%	(E)-Cinnamaldehyde	42.4–89.4%	(E)-Cinnamaldehyde	54.6–90.1%
Eugenol	1.6–13.3%	Eugenyl acetate	1.0–8.1%	(Z)-Cinnamaldehyde	0.6–12.3%	(E)-Cinnamyl acetate	1.4–12.5%
(E)-Cinnamyl acetate	0.3–10.6%	Linalool	0.2–5.0%	(E)-Cinnamyl acetate	0.1–5.4%	(Z)-Cinnamaldehyde	0.4–10.5%
Linalool	0.2–7.0%	(E)-Cinnamyl acetate	0.8–4.6%	Benzaldehyde	0.4–2.3%	Benzaldehyde	1.1–6.3%
beta-Phellandrene	1.5–8%	Benzyl benzoate	trace–4.1%	alpha-Terpineol	trace–2.0%	Eugenol	trace–5.8%
Beta-Caryophyllene	1.3–6.9%	Beta-Caryophyllene	1.9–4.1%	Coumarin	trace–1.9%	Cinnamyl alcohol	0–5.7%
p-Cymene	1.7–4.0%	Safrole	0–1.3%	Salicylaldehyde	0.04–1.8%	Salicylaldehyde	0.05–3.1%
1,8-Cineole	0.4–2.3%	(E)-Cinnamaldehyde	0.6–1.1%	Borneol	trace–1.3%	⍺-copaene	trace–3.0%
Benzaldehyde	trace–2.2%	p-Cymene	0.3–0.8%	Benzyl benzoate	trace–1.0%	Benzyl benzoate	trace–2.9%
alpha-Terpineol	0.4–1.6%	Cinnamyl alcohol	0–0.6%	Cinnamyl alcohol	0–0.04%	Delta-Cadinene	trace–2.6%
Camphor	trace–1.4%	1,8-Cineole	trace–0.6%			Coumarin	0.03–2.5%
		Beta-Phellandrene	0.2–0.5%			Phenylpropanol	trace–1.6%
						⍺-Amorphene	trace–1.1%
						Anisaldehyde	0–1.0%
						(E)-Cinnamic acid	trace–0.9%
						Methyl eugenol	trace–0.1%

**Table 4:** Experimental data for antidiabetic activity of cinnamon oil and some of its main constituents.

Study Product	Study Type	Dosage	Effect	Reference
Cinnamon oil	Animal (Rat), KK-Ay	25, 50, 100 mg/kg b.w.	Significant decrease in fasting blood glucose, plasma C-peptide, serum triglyceride, total cholesterol, and blood urea nitrogen levels, with significant increase in high-density lipoprotein after 35 days. Glucose tolerance was improved and pancreatic islet β-cells showed increased immunoreactivity.	Ping, Zhang, and Ren (2010) [7]
Cinnamon oil (encapsulated emulsion)	Animal (Rat), STZ	200 or 400 mg/kg b.w.	Both doses improved levels of glucose, insulin, SOD, GSH, amylase, lipid profile, and hepatic MDA. Gene expression was modulated to favor antidiabetic outcomes. Positive histological changes seen in liver and pancreas.	Mohammed, Ahmed, Sharaf, El-Nekeety, Abdel-Aziem, Mehaya, Abdel-Wahhab (2020) [35]
Cinnamon oil (encapsulated)	Animal (Rat), STZ	200 or 400 mg/kg b.w.	Treatment with encapsulated cinnamon oil showed improvement in all diabetes-related markers in STZ-treated rats, including liver and kidney function, insulin and glucose levels, lipid profile, and antioxidant enzymes.	Mohammed (2020) [36]
Cinnamon oil	Animal (Rat), Alloxan	5, 10, 20 mg/kg b.w., i.p.	Decreases in fasting blood glucose, total cholesterol, markers of kidney damage and glutathione were observed in treated animals. Histological studies of kidney showed reduced glomerular expansion and tubular dilatations.	Mishra, Bhatti, Singh, Ishar (2010) [37]
Cinnamon oil	Animal (Rat), STZ	100, 200, or 400 mg/kg b.w.	Treatment with cinnamon oil showed significant improvement in histopathology of testicular organs compared to untreated diabetic rats.	Budiastuti, Safitri, Plumeriastuti, Srianto, Effendi (2020) [38]
Cinnamon oil	Human	400 mg/day	Fasting blood glucose levels and insulin levels, along with Quality-of-Life measures, showed improvement after treatment with cinnamon oil, although results were not statistically significant. Pharmacokinetic data indicated low bioavailability.	Stevens (2020) [28]
Cinnamon oil	Animal (Mouse), Balb C	0.2 and 1.0 μL/cage, inhalation	Docking simulations showed interaction of cinnamon oil constituents with leptin receptor in olfactory bulb. In vivo studies confirmed interaction with leptin receptor resulting in decreased appetite and lower weight gain in treated mice.	Kusmardi, Tedjo, Fadilah, Arsianti, Paramita (2018) [39]
Cinnamon oil	Animal (Rat), STZ	5% cinnamon oil in commercial chow	Treatment with cinnamon oil resulted in decreased blood glucose, triglycerides, LDL-cholesterol, and ALT, while levels of HDL-cholesterol were increased compared to diabetic rats.	Zari, Al-Logmani (2009) [40]
Cinnamon oil	Animal (Rat), Alloxan	5, 10, 20 mg/kg b.w., i.p.	Cinnamon oil significantly ameliorated blood glucose levels and thermal hyperalgesia compared to untreated diabetic controls.	Bhatti, Kaur, Singh, Ishar (2009) [41]
Cinnamaldehyde	In vitro (HEK293 and 3T3-L1)	--	Cinnamaldehyde induced expression of peroxisome proliferator-activated (PPAR) genes in 3T3-L1 adipocytes and increased target mRNA expression in HEK293- derived cells.	Li, Futakawa, Yamamoto, Kasahara, Tagami, Liu, and Moriyama (2015) [42]
Cinnamaldehyde	Animal (Mouse), DIO-mice	250 mg/kg/day	Cinnamaldehyde induced significant reduction in cumulative food intake, gastric emptying rates, and ghrelin. Upregulation of genes involved in fatty-acid oxidation was observed in adipose tissue, and mice showed improved glucose tolerance over 5 weeks.	Camacho, Michlig, de Senarclens-Bezencon, Meylan, Meystre, Pezzoli, Markram, le Coutre (2015) [43]
Cinnamaldehyde	Animal (Mouse), db/db	0.02% added to normal chow diet	Treatment with cinnamaldehyde improved aortic tone and function and normalized elevated kidney markers. Treatment also ameliorated glomerular fibrosis and renal dysfunction. Authors suggest a protective effect against vascular dysfunction by inhibiting oxidative stress via Nrf2 signaling pathway activation.	Wang, Yang, Wang, Yang, Wan, Liu, Zhou, Yang (2020) [44]
Cinnamaldehyde	Animal (Rat), STZ	20 mg/kg b.w.	Oral administration led to insulinotropic effects, with increased glucose uptake through GLUT4 receptors and improved function of pyruvate kinase and phosphoenolpyruvate carboxykinase.	Anand, Murali, Tandon, Murthy, Chandra (2010) [45]
Cinnamaldehyde	Animal (Rat), FSD/STZ	20 mg/kg b.w.	Gestating rats treated with cinnamaldehyde showed numerous improvements in health markers compared to diabetic controls, including improved lipid panels and glucose tolerance, more viable fetuses, and improved fetal glucose and insulin levels.	Hosni, Abdel-Moneim, Abdel-Reheim, Mohamed, Helmy (2017) [46]
Cinnamaldehyde	Animal (Rat), FSD/STZ	20 mg/kg b.w.	In rats with gestational diabetes, treatment with cinnamaldehyde prevented development of placental vasculopathy and fetal hypoxia while also alleviating maternal and fetal hyperglycemia.	Hosni, El-Twab, Abdul- Hamid, Prinsen, AbdElgwad, Abdel-Moneim, Beemster (2021) [47]
Cinnamaldehyde	Animal (Mouse), STZ	20 mg/kg/day	Treated mice showed significantly improved insulin sensitivity and glucose metabolism, as well as positive changes in gut microbiota. Authors suggest that modulating host metabolomics may directly or indirectly affect expression levels of genes related to glucose metabolism.	Zhao, Wu, Duan, Liu, Zhu, Zhang, Wang (2021) [48]
Cinnamaldehyde	Animal (Rat), STZ	20 mg/kg/day	Treatment with cinnamaldehyde prevented development of hyperglycemia and insulin resistance following STZ administration.	El-Bassossy, Fahmy, Dadawy (2011) [49]
Cinnamaldehyde	Animal (Rat), STZ	10, 20, 40 mg/kg b.w., p.o.	Rats treated with cinnamaldehyde showed reduced blood glucose levels and amelioration of neurochemical and behavioral deficits seen in diabetic rats. Reductions in IL-2 and TNF-⍺ levels were also noted.	Jawale, Datusalia, Bishnoi, Sharma (2016) [50]
Cinnamaldehyde	Rat	125, 250, 500 mg/kg b.w.	Pharmacokinetic determination of C_max_ in rats administered 125, 250, and 500 mg/kg b.w. cinnamaldehyde was 249, 121, and 82 ng/mL serum, respectively. Estimated half-life of cinnamaldehyde was 6.2–6.9 h.	Zhao, Xie, Yang, Cao, Tu, Cao, Wang (2014) [51]
Eugenol	Animal (Mouse), STZ	100 mg/kg b.w. i.p., 2× per week for 2 weeks	Significant reduction in advanced glycation end-products (AGE) and blood glucose levels.	Singh et al. (2014) [52]
Eugenol	Animal (Rat), STZ		Treatment with eugenol produced lower blood glucose, decrease in serum glycosylated hemoglobin (HbA1C), lipase, and angiotensin-converting enzyme. Lipid panel levels were also positively affected.	Mnafgui et al. (2013) [53]
Eugenol	Animal (Rat), STZ	2.5, 5, 10 mg/kg b.w.	Eugenol improved blood glucose and HbA1C levels in diabetic rats and returned glucose metabolism enzyme levels to near normal. Body weight and liver function also improved.	Srinivasan et al. (2013) [54]
Eugenol	Animal (Rat), FSD/STZ	10 mg/kg b.w.	Levels of fasting blood glucose, insulin, triglyceride, cholesterol, and low-density lipoprotein were all improved. Glutathione levels were increased, as were GLUT4 and AMPK levels in skeletal muscle. Homeostasis model assessment of insulin resistance (HOMA-IR) was significantly lower in rats treated with eugenol compared to diabetic controls.	Al-Trad, Alkhateeb, Alsmadi, Al-Zoubi (2019) [55]
Eugenol	Animal (Rat), HFD	20, 40 mg/kg b.w.	Plasma glucose and insulin levels decreased in a dose-dependent manner, and hepatic gluconeogenesis was inhibited via the CAMKK-AMPK-CREB signaling pathway.	Jeong, Kim, Quan, Jo, Kim, Chung (2014) [56]
Eugenol	Animal (Rat), STZ	5, 10 mg/kg b.w.	Diabetic neuropathy parameters (both blood markers and histological changes) were ameliorated in diabetic rats treated with eugenol. Overexpression of TGF-β1 associated with diabetes was also reduced.	Garud, Kulkarni (2017) [57]
Eugenol	Animal (Rat), STZ	10 mg/kg b.w.	Diabetic rats treated with eugenol showed diminished oxidative stress markers and increased antioxidants. In the brain, levels of acetylcholinesterase and calcium were attenuated. Authors postulate that eugenol may help ameliorate diabetic complications due to oxidative stress.	Prasad, Bharath, Muralidhara (2016) [58]
Eugenol	Animal (Rat), STZ	2 mL/day of a 10% nanoemulsion	Oxidative damage was attenuated, and levels of antioxidants were returned to near-normal levels in diabetic rats compared to untreated controls.	Boroujeni, Dehkordi, Sharifi, Taghian, Mazaheri (2021) [59]
Eugenol	In vitro (Islets of Langerhans cells from male mouse)	50, 100, 200 μM	Total antioxidant capacity, superoxide dismutase, and catalase levels increased in cells treated with eugenol following exposure to hydrogen peroxide to induce oxidative stress. Eugenol can bolster antioxidant systems in islet cells that are particularly vulnerable to oxidative stress in diabetics.	Oroojan, Chenani, An’aam (2020) [60]
Eugenol	Animal (Rat), Alloxan	5, 10, 15 mg/kg b.w.	Diabetic rats treated with eugenol showed lower fasting blood glucose, and improved morphology of liver and islet of Langerhans cells.	Hamdin, Utami, Muliasari, Prasedya, Sudarma (2019) [61]
β-caryophyllene	In silico	--	β-caryophyllene showed affinity for interaction with insulin downstream signaling molecules such as IRS-1, cSrc, and Akt.	Mani, Balraj, Venktsan, Soundrapandiyan, Kasthuri, Danavel, Babu (2021) [62]
β-caryophyllene	Animal (Rat), STZ	10 mg/kg b.w.	Diabetic neuropathy was attenuated in rats treated with β-caryophyllene. Depression behavior and cytokine markers of diabetes were also reduced.	Aguilar-Ávila, Flores-Soto, Tapia-Vázquez, Pastor-Zarandona, López-Roa, Viveros-Paredes (2019) [63]
β-caryophyllene	Animal (Rat), STZ	200 mg/kg b.w.	Hyperglycemia was attenuated by treatment with β-caryophyllene, and oxidative stress was avoided through increased activity of antioxidant enzymes.	Basha, Sankaranarayanan (2016) [64]
β-caryophyllene	Animal (Rat), STZ	200 mg/kg b.w.	Plasma insulin levels were rescued to near-normal levels in diabetic rats treated with β-caryophyllene.	Basha, Sankaranarayanan (2015) [65]
β-caryophyllene	Animal (Rat), STZ	100, 200, 400 mg/kg b.w.	Administration of β-caryophyllene ameliorated STZ-induced changes in blood glucose, insulin levels, and glucose metabolism enzymes. The antidiabetic and insulinotropic effects were most pronounced at the 200 mg/kg dose.	Basha, Sankaranarayanan (2014) [66]
β-caryophyllene	Animal (Rat), HFD	30 mg/kg b.w.	Treatment with β-caryophyllene improved glycemic and lipidemic markers and reduced vascular oxidative stress and inflammation.	Youssef, El-Fayoumi, Mahmoud (2019) [67]
β-caryophyllene	In vitro (mesangial cells)	--	β-caryophyllene modulated NF-κB and Nrf pathways and exhibited anti-inflammatory and nephroprotective activity in mesangial cells under high-glucose conditions.	Li, Wang, Chen, Yang (2020) [68]
β-caryophyllene	In vitro (C2C12 myotubes)	--	β-caryophyllene significantly increased skeletal muscle uptake of glucose and glycolytic production of ATP through cannabinoid receptor-2-mediated pathways.	Geddo et al. (2021) [69]

**Table 5:** Binding affinities of cinnamon essential oil constituents against selected glucose metabolism enzymes (all results in units of kcal/mol).

**Essential Oil Ligand**	**PTP1B**	**α-Glucosidase**	**Glucokinase**	**α-Amylase**	**HK-II**
β-caryophyllene	−5.6	−6.6	−6.3	−6.9	−6.6
Cinnamaldehyde	−6.5	−6.1	−5.8	−5.4	−5.5
Eugenol	−6.0	−6.7	−6.0	−5.6	−5.6
**Positive Controls**	**PTP1B**	**α-Glucosidase**	**Glucokinase**	**α-Amylase**	**HK-II**
Acarbose	--	−8.3	--	−7.3	--
Piragliatin	--	--	−9.5	--	--
TPICOOH	−9.7	--	--	--	--
Benserazide	--	--	--	--	−7.4

## Supplementary Materials

The following are available online. Figure S1: Molecular modeling of cinnamon essential oil constituents docked with alpha-amylase. (A) Alpha-amylase antagonist acarbose and essential oil compounds cinnamaldehyde, beta-caryophyllene, and eugenol docked in the active site of alpha-amylase. (B) Close view of alpha-amylase antagonist acarbose and essential oil compounds cinnamaldehyde, beta-caryophyllene, and eugenol docked in the active site of alpha-amylase, specifically near residues ASP197, GLU233, and ASP300. (C) Acarbose docked in the active pocket of alpha-amylase, with a binding affinity of −7.3 kcal/mol. (D) Cinnamaldehyde docked in the active pocket of alpha-amylase, with a binding affinity of −5.4 kcal/mol. (E) Beta-caryophyllene docked in the active pocket of alpha-amylase, with a binding affinity of −6.9 kcal/mol. (F) Eugenol docked in the active pocket of alpha-amylase, with a binding affinity of −5.6 kcal/mol. Figure S2: Molecular modeling of cinnamon essential oil constituents docked with glucokinase. (A) Glucokinase agonist piragliatin and essential oil compounds cinnamaldehyde, beta-caryophyllene, and eugenol docked in the active site of glucokinase. (B) Close view of glucokinase agonist piragliatin and essential oil compounds cinnamaldehyde, beta-caryophyllene, and eugenol docked in the active site of alpha-glucosidase, specifically near residues VAL 62, ARG 63, TRP 99, TYR 214, MET 235, LYS 458, LYS 459, and LEU 451. (C) Piragliatin docked in the active pocket of glucokinase, with a binding affinity of −9.5 kcal/mol. (D) Cinnamaldehyde docked in the active pocket of glucokinase, with a binding affinity of −5.8 kcal/mol. (E) Beta-caryophyllene docked in the active pocket of glucokinase, with a binding affinity of −6.3 kcal/mol. (F) Eugenol docked in the active pocket of glucokinase, with a binding affinity of −6.0 kcal/mol. Figure S3: Molecular modeling of cinnamon essential oil constituents docked with hexokinase-II (HK-II). (A) HK-II antagonist benserazide and essential oil compounds cinnamaldehyde, beta-caryophyllene, and eugenol docked in the active site of HK-II. (B) Close view of HK-II antagonist benserazide and essential oil compounds cinnamaldehyde, beta-caryophyllene, and eugenol docked in the active site of HK-II, specifically near residues ASN656, THR680, and GLY681. (C) Benserazide docked in the active pocket of HK-II, with a binding affinity of −7.4 kcal/mol. (D) Cinnamaldehyde docked in the active pocket of HK-II, with a binding affinity of −5.5 kcal/mol. (E) Beta-caryophyllene docked in the active pocket of HK-II, with a binding affinity of −6.6 kcal/mol. (F) Eugenol docked in the active pocket of HK-II, with a binding affinity of −5.6 kcal/mol.

## Sample Availability

Samples of the compounds are not available from the authors.
