# Supplementary material for: Antidiabetic Potential of Volatile Cinnamon Oil: A Review and Exploration of Mechanisms Using In Silico Molecular Docking Simulations
Source: Molecules. 2022 Jan 27;27(3):853. doi: 10.3390/molecules27030853 (PMC8840343; doi:10.3390/molecules27030853)
Supplement: Supplementary file 1 [file molecules-27-00853-s001.zip › molecules-1545229-Supplemental Material.pdf]

Supplemental Material

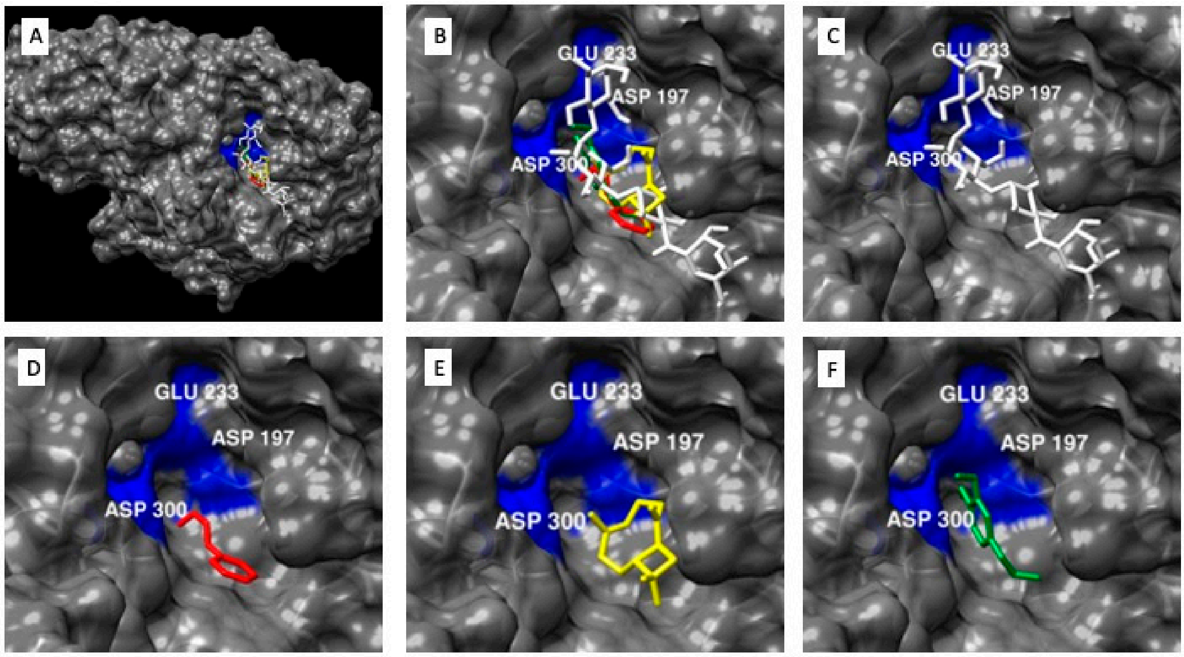

Figure S1. Molecular modeling of cinnamon essential oil constituents docked with alpha-amylase. (**A**) Alpha-amylase antagonist acarbose and essential oil compounds cinnamaldehyde, beta-caryophyllene, and eugenol docked in the active site of alpha-amylase; (**B**) Close view of alpha-amylase antagonist acarbose and essential oil compounds cinnamaldehyde, beta-caryophyllene, and eugenol docked in the active site of alpha-amylase, specifically near residues ASP197, GLU233, and ASP300; (**C**): Acarbose docked in the active pocket of alpha-amylase, with a binding affinity of −7.3 kcal/mol; (**D**) Cinnamaldehyde docked in the active pocket of alpha-amylase, with a binding affinity of −5.4 kcal/mol; (**E**) Beta-caryophyllene docked in the active pocket of alpha-amylase, with a binding affinity of −6.9 kcal/mol; (**F**) Eugenol docked in the active pocket of alpha-amylase, with a binding affinity of −5.6 kcal/mol.

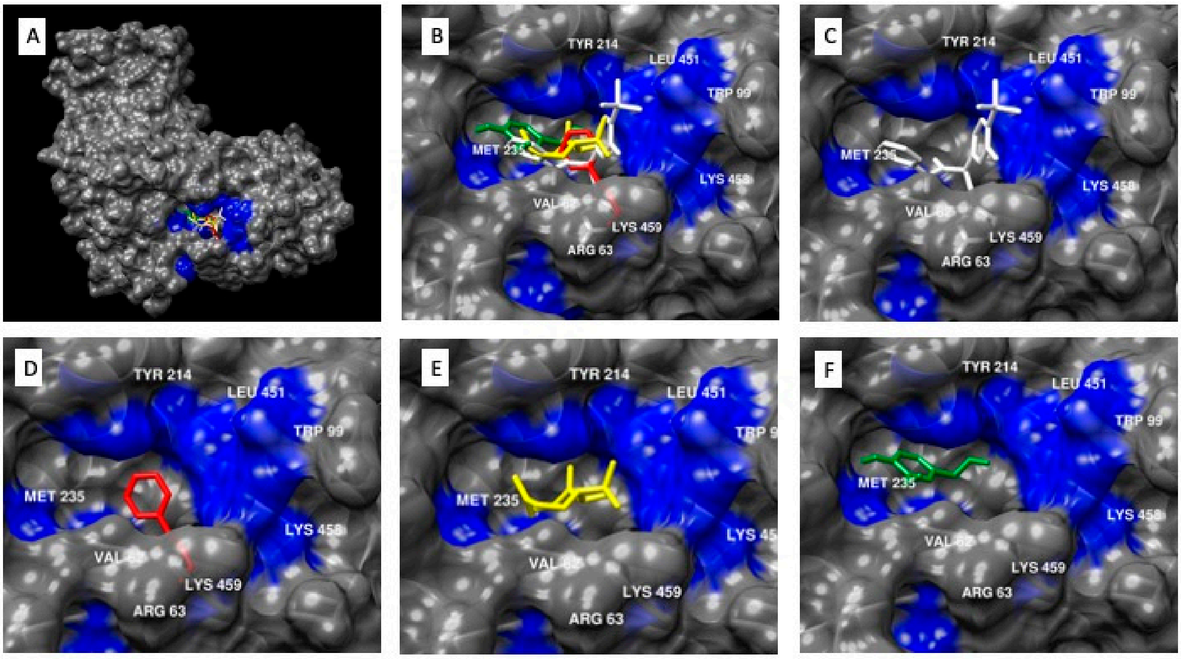

Figure S2. Molecular modeling of cinnamon essential oil constituents docked with glucokinase. (**A**) Glucokinase agonist piragliatin and essential oil compounds cinnamaldehyde, beta-caryophyllene, and eugenol docked in the active site of glucokinase; (**B**) Close view of glucokinase agonist piragliatin and essential oil compounds cinnamaldehyde, beta-caryophyllene, and eugenol docked in the active site of alpha-glucosidase, specifically near residues VAL 62, ARG 63, TRP 99, TYR 214, MET 235, LYS 458, LYS 459, and LEU 451; (**C**) Piragliatin docked in the active pocket of glucokinase, with a binding affinity of −9.5 kcal/mol; (**D**) Cinnamaldehyde docked in the active pocket of glucokinase, with a binding affinity of −5.8 kcal/mol; (**E**) Beta-caryophyllene docked in the active pocket of glucokinase, with a binding affinity of −6.3 kcal/mol; (**F**) Eugenol docked in the active pocket of glucokinase, with a binding affinity of −6.0 kcal/mol.

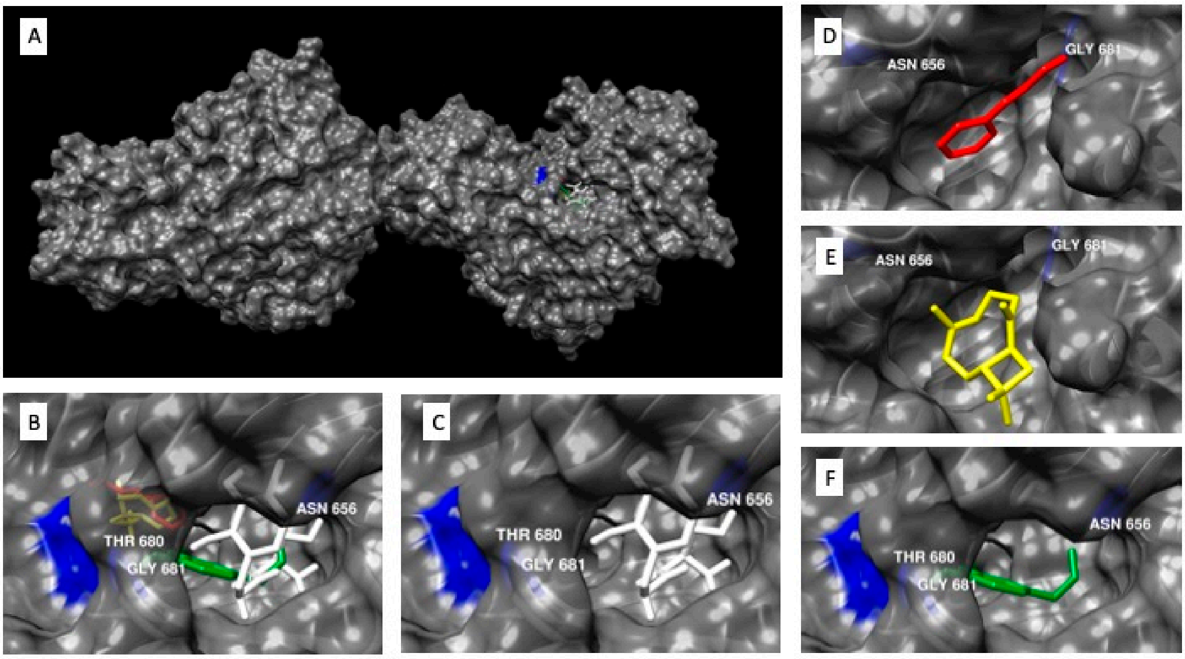

Figure S3. Molecular modeling of cinnamon essential oil constituents docked with hexokinase-II (HK-II). (**A**) HK-II antagonist benserazide and essential oil compounds cinnamaldehyde, beta-caryophyllene, and eugenol docked in the active site of HK-II; (**B**) Close view of HK-II antagonist benserazide and essential oil compounds cinnamaldehyde, beta-caryophyllene, and eugenol docked in the active site of HK-II, specifically near residues ASN656, THR680, and GLY681; (**C**) Benserazide docked in the active pocket of HK-II, with a binding affinity of −7.4 kcal/mol; (**D**) Cinnamaldehyde docked in the active pocket of HK-II, with a binding affinity of −5.5 kcal/mol; (**E**) Beta-caryophyllene docked in the active pocket of HK-II, with a binding affinity of −6.6 kcal/mol; (**F**) Eugenol docked in the active pocket of HK-II, with a binding affinity of −5.6 kcal/mol.
